# A distinct strain of tomato leaf curl New Delhi virus that causes mosaic disease in ash gourd and other cucurbitaceous crops

**DOI:** 10.3389/fmicb.2023.1268333

**Published:** 2023-10-26

**Authors:** S. Vignesh, P. Renukadevi, K. Nagendran, N. Senthil, R. Vinoth Kumar, R. SwarnaPriya, Tusar Kanti Behera, G. Karthikeyan

**Affiliations:** ^1^Department of Plant Pathology, Tamil Nadu Agricultural University, Coimbatore, Tamil Nadu, India; ^2^Indian Institute of Vegetable Research, Varanasi, Uttar Pradesh, India; ^3^Department of Biotechnology, Tamil Nadu Agricultural University, Coimbatore, Tamil Nadu, India; ^4^Department of Biotechnology, College of Science and Humanities, SRM Institute of Science and Technology, Chennai, Tamil Nadu, India; ^5^Floriculture Research Station, Tamil Nadu Agricultural University, Coimbatore, Tamil Nadu, India

**Keywords:** begomovirus, pathogenesis, evolution, agro-inoculation, cucurbits, qPCR

## Abstract

Ash gourd (*Benincasa hispida*) is a cucurbitaceous crop cultivated as an edible vegetable rich in vitamins, minerals, dietary fibers and antioxidants. In a field survey conducted in the Udumalpet region of Tamil Nadu during 2019, the incidence of mosaic disease on ash gourd crop was observed to be 75%. The DNA-A and DNA-B components of begomovirus genome have been identified as associated with this disease. Both the cloned DNA-A and DNA-B genomic components shared highest pairwise sequence identities with the isolates of tomato leaf curl New Delhi virus (ToLCNDV), a bipartite begomovirus. Recombinant analysis showed that both the components are possibly evolved through intra-species recombination between ToLCNDV isolates. Tomato leaf curl Bangladesh betasatellite (ToLCBB) is not naturally associated with this sample. The results of infectivity studies on ash gourd and other cucurbitaceous crops demonstrates the Koch’s postulates, when co-inoculation of DNA-A and DNA-B of ToLCNDV was undertaken. However, the inoculation of non-cognate ToLCBB along with DNA-A and DNA-B enhances the symptom expression and reduces the time taken for symptom development. Thus, Koch’s postulates were proved for these virus complexes on cucurbitaceous crops. Furthermore, an enhanced accumulation of DNA-A component was detected in the cucurbits co-inoculated with ToLCNDV and ToLCBB. This report highlights the importance of investigating the spread of these disease complexes with other cucurbitaceous crops in India.

## 1. Introduction

Cucurbits are an economically important crop grown in an area of around 7.89 million hectares with a global production of around 238.6 million tonnes. Among the different cucurbits, ash gourd [*Benincasa hispida* (Thunb.) Cogn.] is an important vegetable and unique melon mainly grown in India for its edible fruit. Fruits are not only consumed as a vegetable but also used in the confectionary industry for making candy, jam, ketchup, and cakes, among other dishes. The ash gourd crop is cultivated around the year both in tropical and subtropical regions of the Indian subcontinent. Viral infections of the plant pose a major threat to its productivity and the quality of the cucurbitaceous crops (Thresh, 2006; Damicone et al., 2007). Several viruses including begomoviruses (Riyaz et al., 2013; Roy et al., 2013; Kumari et al., 2022), potyviruses (Nagendran et al., 2017a), and tobamoviruses (Kumar et al., 2017) have been identified in ash gourd in India.

The Begomovirus (Family: Geminiviridae) comprises more than 450 species and causes diseases in a wide range of economically important crops as well as weeds in the “Old World” and “New World” regions (Brown et al., 2015; Kumar, 2019). Begomoviruses are transmitted by whiteflies (*Bemisia tabaci*) in a persistent and circulative manner. They are further subdivided into either bipartite consisting of two approximately similar sized DNA-A and DNA-B genomic components or monopartite with a homologous DNA-A-like genomic component. The DNA-A component encodes six proteins necessary for viral replication and encapsidation, whereas DNA-B has two proteins that mediate symptom expression and systemic virus movement (Kumar, 2019). These begomoviral genomic components were often associated with betasatellites to promote pathogenesis, virulence, and establishment of characteristic disease symptoms (Rouhibakhsh and Malathi, 2005; Sivalingam and Varma, 2012; Jyothsna et al., 2013; Ranjan et al., 2014; Kumar et al., 2015; Sharma et al., 2019; Singh et al., 2021). Begomoviruses undergo rapid evolution through mutation and recombination (Lefeuvre et al., 2007; Prasanna and Rai, 2007; Melgarejo et al., 2013; George et al., 2015; Kumar et al., 2015, 2017).

The whitefly-transmitted Tomato leaf curl New Delhi virus (ToLCNDV) is a limiting factor for cucurbit production in Asian and European countries (Srivastava et al., 1995; Hussain et al., 2004; Maruthi et al., 2005; Chang et al., 2010; Sangeetha et al., 2018). It has become a significant threat to numerous crops across plant families including the *Apocyanaceae, Acanthaceae, Asteraceae, Cucurbitaceae, Caricaceae, Euphorbiaceae, Fabaceae, Malvaceae, Papaveraceae, Phyllanthaceae,* and *Solanaceae* worldwide (Kumar et al., 2015; Moriones et al., 2017; Zaidi et al., 2017; Nagendran et al., 2017b; Ashwathappa et al., 2020; Gemert et al., 2020; Chakraborty and Kumar, 2021; Krishnan et al., 2023). The infection of ToLCNDV on cucurbits exhibits characteristic symptoms including yellow mosaic, reduction of internodal length, severe curling, vein swelling, rough skin, reduced fruit setting, etc. (Moriones et al., 2017; Zaidi et al., 2017). In this study, we have characterized the association of ToLCNDV infecting ash gourd from Tamil Nadu, India. The infectivity study highlighted the effect of either cognate DNA-B or non-cognate betasatellite for disease development on various cucurbitaceous crops.

## 2. Materials and methods

### 2.1. Survey and sample collection

Ash gourd leaf samples showing yellow mosaic, leaf puckering, chlorosis, and severe yellow mosaic symptoms were collected from the agricultural fields in the Udumalpet region (GPS co-ordinates: 10° 35′6.282″ N, 77°14′52.476″ E), a major ash gourd growing area of Tamil Nadu during November 2019. Totally eight samples from four different fields along with one apparently healthy sample were collected and stored at −20°C for further studies.

### 2.2. Detection of begomovirus genomic components

The total genomic DNA was isolated from all the ash gourd leaf samples using the CTAB method (Doyle and Doyle, 1990). The total DNAs were subjected to polymerase chain reaction (PCR) assay using the degenerate primers, PAR1v722/PAL1c1960 (Table 1) amplifying ~1.2 kb on DNA-A genome of begomovirus (Chatchawankanphanich and Maxwell, 2002).

**Table 1:** Details of primer sequences used in this study.

Primer ID	5′–3′ sequence	Target virus	Amplicon size	References
PAL1c1960	ACNGGNAARACNATGTGGGC	Begomovirus	~ 1,200 bp	Chatchawankanphanich and Maxwell (2002)
PAR1v722	GGNAARATHTGGATGGA			
UN101	AAGCTTGCGACTATTGTATGAAAGAGG	Alphasatellite	~1,350 bp	Bull et al. (2003)
UN102	AAGCTTCGTCTGTCTTACGAGCTCGCTG			
Beta01	GGTACCACTACGCTACGCAGCAGCC	Betasatellite	~1,350 bp	Briddon et al. (2002)
Beta02	GGTACCTACCCTCCCAGGGGTACAC			
GKNDV DNA-A-F	CGCAGGTTGTGGTTGAACTG	ToLCNDV DNA-A	~630 bp	This study
GKNDV DNA-A-R	GCAAAACAATGTGGGCTCGT			
GKNDV DNA-B-F	TCCAACAGTGGTCCCCATCT	ToLCNDV DNA-B	~350 bp	This study
GKNDV DNA-B-R	GCCCTTGTTTCCGTTGTACG			
GKNDVqCP-F	AGAAGTCCAGACGTGCCAAG	ToLCNDV-CP	~120 bp	This study
GKNDVqCP-R	CGGTTCCACGGGTAACATCA			

### 2.3. Rolling circle amplification and cloning

Seventy to eighty ng of total DNA from all the PCR positive samples were subjected to rolling circle amplification (RCA) as per the manufacturer’s instructions (Thermo Fisher Scientific, United States; Supplementary Figure 2). The resultant RCA product was digested individually with five restriction endonucleases (*Bam*HI, *Hind*III, *Kpn*I, *Xba*I, and *Eco*RI; Supplementary Figure 3). The ~2.7 kb fragments were gel purified and cloned into a pUC18 vector (Thermo Fisher Scientific, United States) already linearized with the corresponding restriction endonucleases. The recombinant clones were screened by restriction analysis. The predicted full-length genome fragments were selected and sequenced using primer walking with M/S. Eurofins Genomics, Bangalore, India. A separate PCR assay was also done to examine RCA products for the association of alpha-satellites and beta-satellites with universal primer pairs, UN101/UN102 and Beta01/Beta02 (Table 1; Briddon et al., 2002; Bull et al., 2003).

### 2.4. Sequence analysis

The cloned viral genome sequences of the DNA-A (pUCND-A) and DNA-B (pUCND-B) with the previously reported isolates available in the NCBI database were aligned using the MUSCLE algorithm (Edgar, 2004). The Sequence Demarcation Tool (SDT) version 1.2 was used to assess the pairwise sequence identity among the viral sequences (Muhire et al., 2014). The maximum likelihood-based phylogenetic trees were generated using MEGA 11 software (Kumar et al., 2016). Using RDP4 software (Martin et al., 2015), the recombination analysis was performed as described earlier (George et al., 2015; Martin et al., 2015).

### 2.5. Construction of agro-infectious constructs

Partial tandem repeat DNA of the cloned viral genomic components were constructed to satisfy Koch’s postulates. A 750 bp fragment of pUCND-A [*Bam*HI (125)-*Pst*I (2110)] containing intergenic region (IR) was cloned into pCAMBIA2301 vector linearized with *Bam*HI and *Pst*I. The full-length monomer linearized with *Bam*HI was then ligated to generate a tandem repeat construct of pUCND-A (referred to as A). For pUCND-B, a 2.1 kb [*Kpn*I (1532)-*Bam*HI (925)] fragment was cloned into pCAMBIA2301, followed by insertion of the full-length monomer (~2.7) linearized with *Kpn*I was then ligated to generate tandem repeat construct of pUCND-B (referred as B; Supplementary Figures 4–7). Further, a dimeric construct of tomato leaf curl Bangladesh betasatellite (ToLCBB; OQ718502) available in our laboratory was also used (referred to as β).

### 2.6. Agro-inoculation

The tandem repeat plasmid constructs of DNA-A and DNA-B were mobilized into *Agrobacterium tumefaciens* strain GV3101 (Supplementary Figure 8) by freeze–thaw technique (Chen et al., 1994). Agro-inoculation was performed on 10-days old ash gourd (cv. Suruchi), squash (cv. Green star), pumpkin (cv. Co2), bitter gourd (cv. Co1), ridge gourd (cv. CoH1), snake gourd (cv. Co2), cucumber (cv. Nazia F1), muskmelon (cv. Dulce F1), watermelon (cv. F1 555) and bottle gourd (cv. Co1) plants as described by Singh et al. (2021). Plant inoculation with agrobacterium carrying empty pCAMBIA2301 was considered a mock inoculation. After agro-inoculation, the plants were maintained at 28°C ± 2°C in an insect-proof growth chamber with 16/8 h of photoperiod.

### 2.7. Quantification of viral genomic components

The presence of DNA-A, DNA-B, and DNA-β components in the systemic leaves of agro-inoculated plants were analyzed with viral gene specific primers (Table 1). The viral genomic components in the agro-inoculated plants were quantified by quantitative PCR (qPCR). The qPCR was carried out in a CFX-96 real-time system (Bio-Rad) with a 20 μL reaction mixture consisting of 10 μL KAPA SYBR green fast qPCR Master Mix 2X (Sigma-Aldrich), 1.5 μL (10 pmol) of each forward and reverse primer and 2 μL template DNA (~36.5 ng). The optimized thermal cycling reactions were 94°C for 5 min, 40 cycles of 94°C for 30 s, 58°C for 30 s, and 72°C for 30s. All the reactions were performed triplicate in high-profile 96 well qPCR plates (Bio-Rad). The melt curve analysis was programmed from 65 to 95°C, with an increase of 0.5°C at every 5 s interval.

The total genomic DNA isolated from the systemic leaves of agro-inoculated plants were subjected to viral DNA detection using the partial coat protein specific primer. Real-time PCR was carried out using 10-fold serially diluted plasmid DNA as a template for the above. To construct the standard curves for plasmid DNA, a linear regression curve was plotted with the mean Ct values on the Y-axis and the log DNA dilution in ng on the X-axis.

To quantify the ToLCNDV DNA-A titer, systemically infected leaves were collected and total genomic DNA was isolated as described above. The absolute quantification of infectious viral copies were derived by the mean Ct values were fitted into the corresponding standard curve and the viral copy number was calculated by using the formula N = (X ng * 6.0221 × 10^23^ molecules/mole)/(n * 660 g/mole * 1 × 10^9^ ng/g), where N indicates the number of viral copies; X represents the amount of amplicon in ng and n represents the number of bases of the recombinant plasmid (Roy et al., 2021).

### 2.8. Statistical analysis

Data analyses were performed in the R statistical software (R Core Team, 2023). To determine the statistical significance of ToLCNDV accumulation in agro-inoculated cucurbits samples at 28 dpi the mean ± standard error was calculated. The amount of the mock, A, A + B, and A + B + β components were compared using an ANOVA *F*-test (Hothorn et al., 2008).

## 3. Results

### 3.1. Symptomatology and diagnosis

The average disease incidence was 75% in the farmer’s field (data not shown). Based on the symptomatology and the presence of whiteflies in the surveyed fields, the samples were suspected of begomovirus infection.

### 3.2. Association of ToLCNDV with the mosaic disease of ash gourd

**Figure 1:**
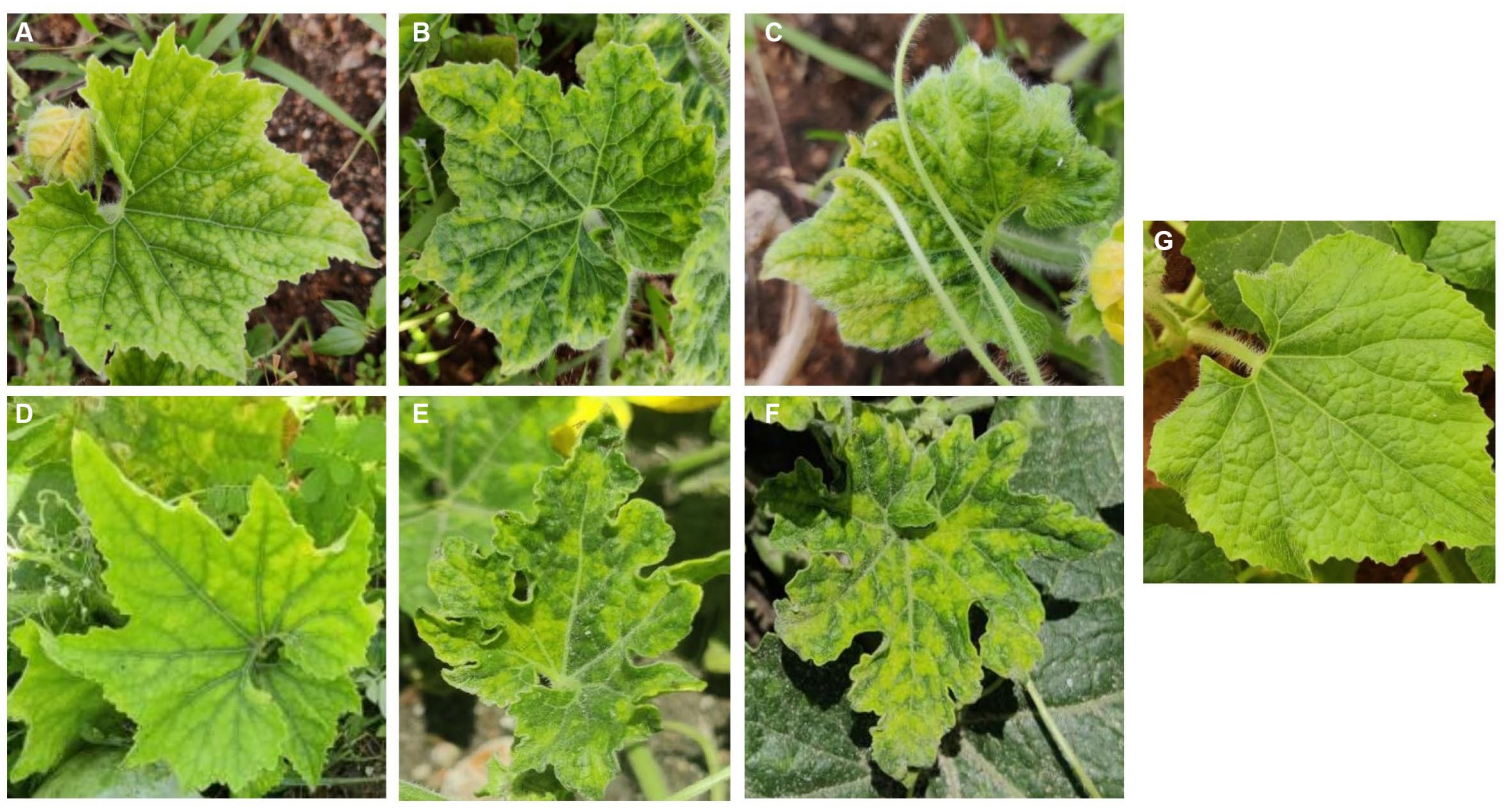
Field symptoms of begomovirus infection on ash gourd. **(A)** Yellow mosaic; **(B)** Mosaic with puckering; **(C)** Yellow mosaic with downward cupping; **(D)** Chlorosis; **(E)** Leaf puckering; **(F)** Severe yellow mosaic pattern; **(G)** Healthy leaf.

The ash gourd plants bearing disease symptoms such as upward and downward cupping of leaves, yellow mosaic, chlorosis, leaf puckering, leaf curl, and malformation of fruits were observed in the cultivated fields at Udumalpet taluk of Tamil Nadu in India in 2019 (Figure 1). The preliminary diagnostic evaluation for begomovirus made through PCR assay using begomovirus universal primer pair (PAL1v722/PAL1c1960) has yielded an expected amplicon of ~1.2 kb from all the symptomatic samples (Supplementary Figure 1). However, these samples tested negative for the association of alphasatellite (UN101/UN102) and betasatellites (Beta01/Beta02) in the PCR assay.

**Figure 2:**
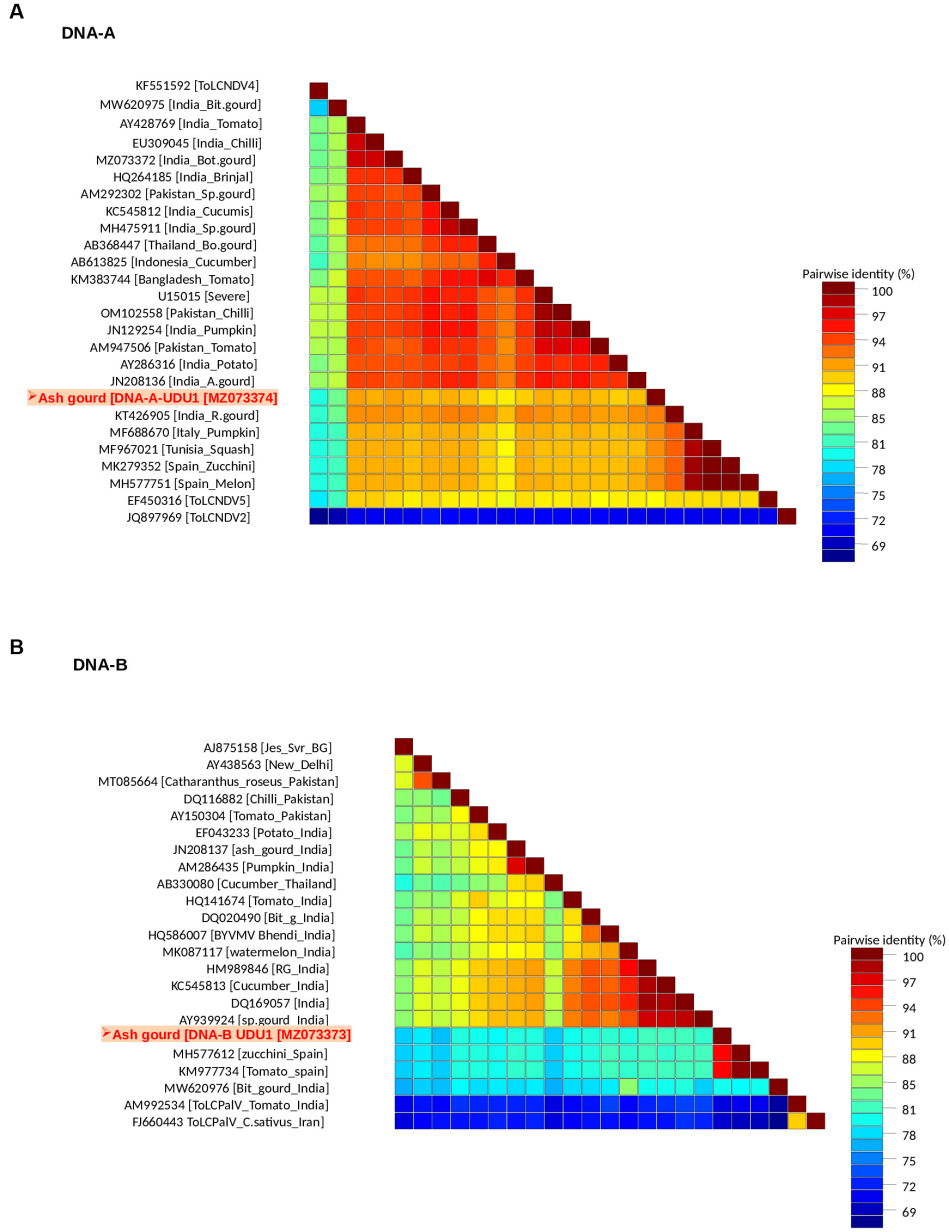
Association of a bipartite begomovirus, ToLCNDV, with the mosaic disease of ash gourd in India. Pairwise sequence identity matrices of begomovirus components, DNA-A **(A)** and DNA-B **(B)**.

**Figure 3:**
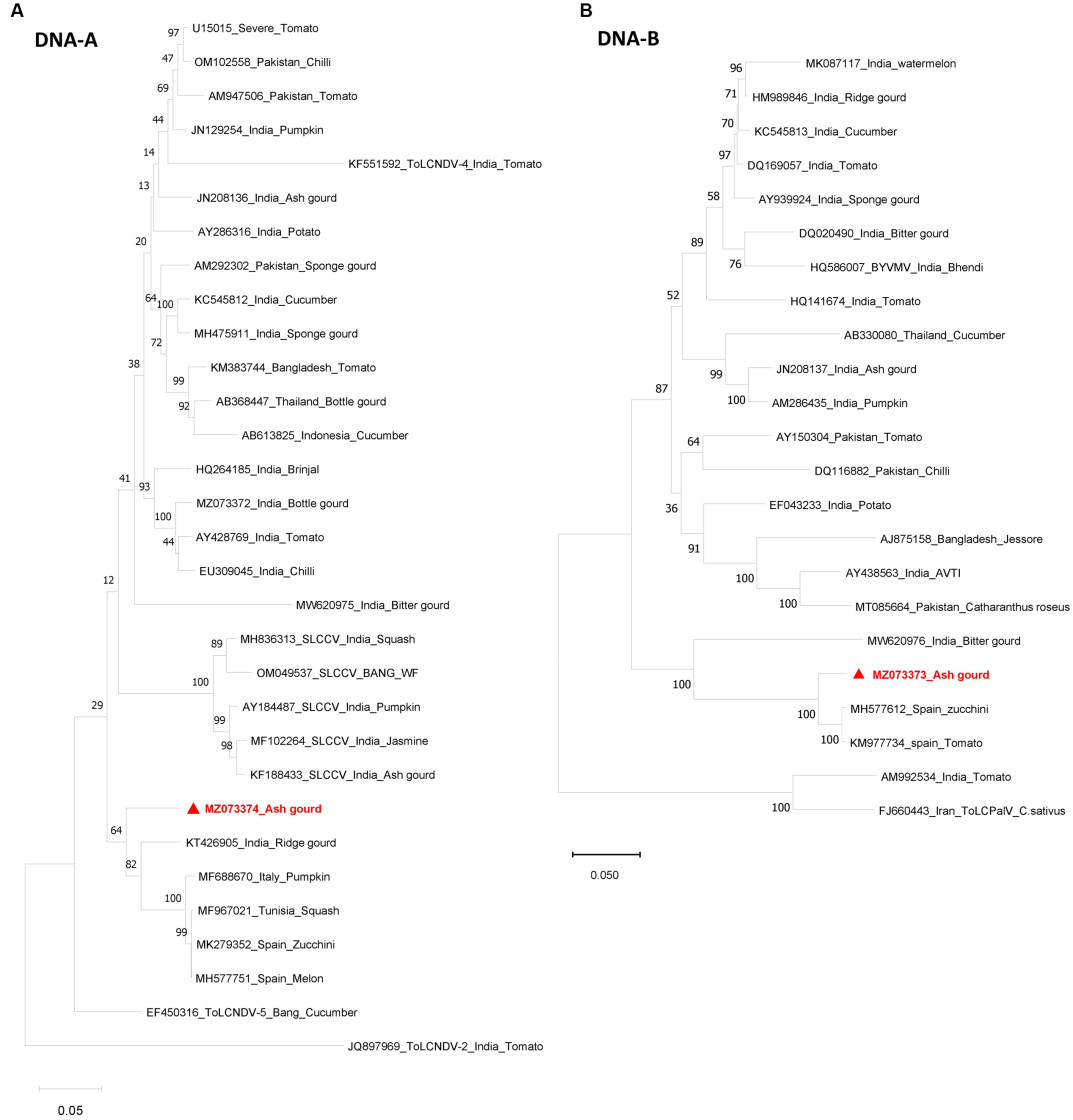
Phylogenetic dendrograms of DNA-A **(A)** and DNA-B **(B)** identified in this study. The numbers at the nodes indicate the percentage bootstrap values of 1,000 replicates. The scale bars represent the genetic distance. Viral genomes identified in this study are marked in red color.

Sequence analysis revealed the presence of both DNA-A (*Bam*HI clone) and DNA-B (*Kpn*I clone). The DNA-A sequence (MZ073374) identified here shared the highest pairwise sequence identity of 92.4% with a ridge gourd isolate (KT426905) of ToLCNDV from India (Figure 2A). Similarly, the DNA-B sequence (MZ073373) showed the highest nucleotide identity of 96.1% with the ToLCNDV isolate infecting zucchini (MH577612) from Spain (Figure 2B). According to begomovirus species demarcation guidelines of 91% (Brown et al., 2015), the cloned begomovirus can be considered as a distinct strain of ToLCNDV. In the phylogenetic dendrogram, this cloned DNA-A component from ash gourd was found to be clustered along with the isolates of ToLCNDV-5 species. In the case of DNA B, the ash gourd isolate had grouped with Spain (tomato and zucchini) isolates in the phylogenetic dendrogram (Figure 3).

### 3.3. Recombination of ToLCNDV

Recombination analysis of the cloned ToLCNDV isolates revealed that the nucleotide sequence of the DNA-A component had evolved through intra-specific recombination between two ToLCNDV-tomato isolates as a major parent and a ToLCNDV-ridge gourd isolate as a minor parent with a breakpoint between 1,521–2,738 nucleotide positions. Similarly, DNA-B showed recombination between ToLCNDV isolate from a ridge gourd as a major parent and a sponge gourd as a minor parent (Table 2).

**Table 2:** Recombination analysis on ToLCNDV genome of ash gourd isolate.

Viral sequence	Break point positions (in nt)	Putative major parent	Putative minor parent	RDP	GC	BS	MC	CHI	SS	3Seq
DNA-A	1,521–2,738 (AC1, IR)	JQ897969; ToLCNDV-Tomato	KT426905; ToLCNDV-Ridge gourd	2.914 × 10^−77^	3.120 × 10^−153^	3.955 × 10^−150^	1.267 × 10^−62^	4.124 × 10^−20^	-	1.154 × 10^−12^
DNA-B	1,254–2,370 (BC1, IR)	HM989846; ToLCNDV-Ridge gourd	AY939924; ToLCNDV-Sponge gourd	1.204 × 10^−181^	1.835 × 10^−175^	-	3.186 × 10^−64^	-	6.895 × 10^−89^	5.107 × 10^−13^

Recombination detection methods abbreviated are GC-GENCONV; BS-BOOTSCAN; MC-MAXCHI; CHI-CHIMERA; SS-SISCAN.

### 3.4. Infectivity assay of the cloned infectious constructs

**Figure 4:**
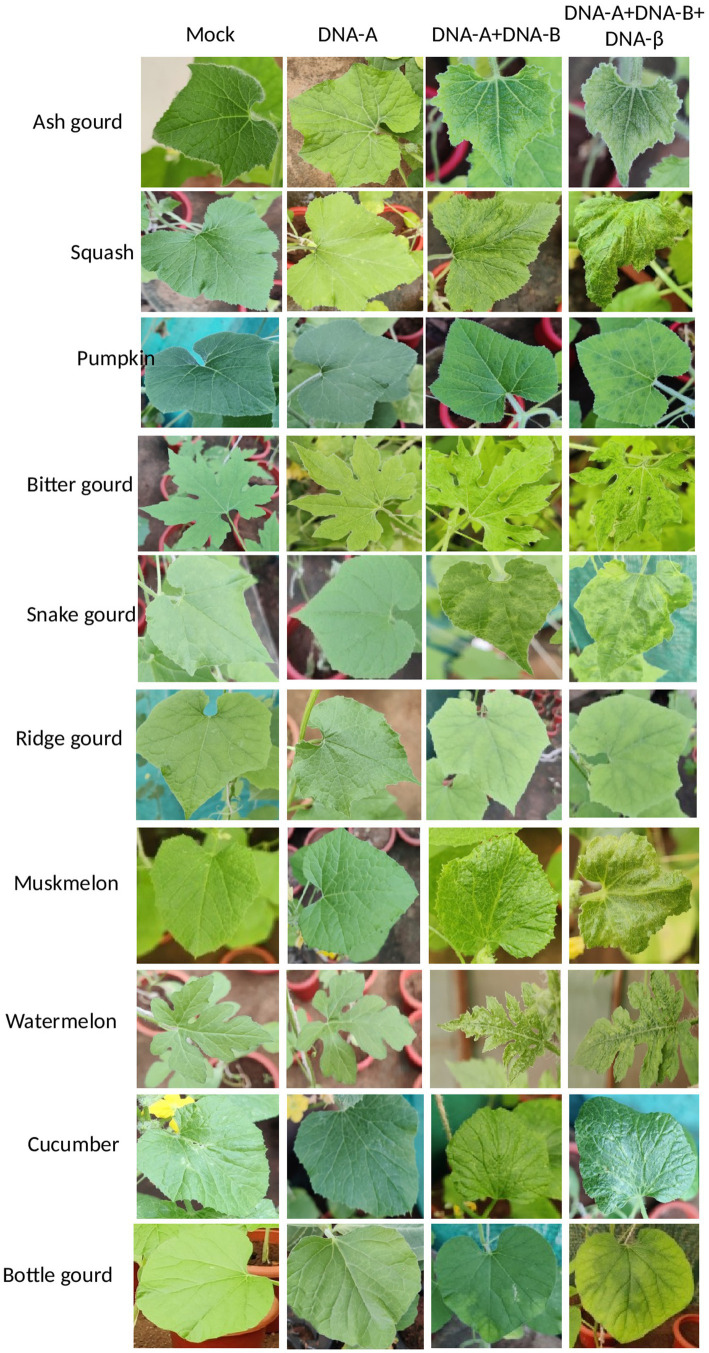
The cloned infectious viral constructs cause disease on various cucurbitaceous crops. The symptom appearance on the agro-inoculated cucurbits is at 28 dpi.

To ascertain the infectivity nature of the cloned viral infectious constructs, 10 different cucurbit plants (ash gourd, squash, pumpkin, bitter gourd, ridge gourd, snake gourd, cucumber, muskmelon, watermelon, and bottle gourd) were used for agro-inoculation study (Supplementary Figure 9). No symptoms were observed on any of the plants inoculated with DNA-A alone till 28 days post inoculation (dpi). However, when the bottle gourd, bitter gourd, cucumber, ridge gourd, and pumpkin plants were co-inoculated with DNA-A and DNA-B, mild mosaic symptoms were noticed (Figure 4). However, severe mosaic symptoms were observed on ash gourd, muskmelon, snake gourd, squash, and watermelon plants when co-inoculated with DNA-A and DNA-B. Interestingly, severe symptoms were observed on all the cucurbits species co-inoculated with DNA-A, DNA-B, and ToLCBB (Figure 4). Moreover, an additional symptom of leaf curling was developed on the bitter gourd, muskmelon, and squash plants co-inoculated with DNA-A, DNA-B, and ToLCBB. In addition to the elevated symptom severity, the reduced incubation period (of 2–4 dpi) and stunting were observed on the plants co-inoculated with DNA-A, DNA-B, and ToLCBB compared to DNA-A and DNA-B co-inoculated plants (Table 3). This phenomenon was noticed in all the cucurbits irrespective of the plant species tested (Supplementary Figure 10). The rate of infection on ash gourd and squash was found to be 100%, whereas the infectivity rate ranged between 45% and 85% on the other cucurbit crops (Table 3).

**Table 3:** Infectivity assay of viral infectious constructs on cucurbitaceous plants.

Host	Inoculum	Latent period (in dpi)	Symptom appearance	Number of symptomatic plants/inoculated plants	PCR positive plants/INOCULATED plants	Disease development and virus detection (by PCR) in %
Ash gourd (cv. Suruchi)	A + B	23	Mosaic, vein banding	16/20	16/20	80
	A + B + β	21	Mosaic, vein banding, stunting	20/20	20/20	100
	A	-	-	0/20	0/20	0
	Mock inoculated control	-	-	0/20	0/20	0
Squash (cv. Green star)	A + B	20	Mosaic, yellow patches	20/20	20/20	100
	A + B + β	20	Mosaic, downward leaf curling with puckering, stunting, yellow patches	20/20	20/20	100
	A	-	-	0/20	0/20	0
	Mock inoculated control	-	-	0/20	0/20	0
Pumpkin (cv. Co2)	A + B	25	Mild mosaic, mild yellowing	9/20	9/20	45
	A + B + β	23	Mosaic, yellowing with dark green patches, stunting	14/20	14/20	70
	A	-	-	0/20	0/20	0
	Mock inoculated control	-	-	0/20	0/20	0
Snake gourd (cv. Co2)	A + B	22	Mosaic, yellowing with dark green patches`	12/20	12/20	60
	A + B + β	21	Mosaic, yellowing with dark green patches, stunting	16/20	16/20	80
	A	-	-	0/20	0/20	0
	Mock inoculated control	-	-	0/20	0/20	0
Ridge gourd (cv. CoH1)	A + B	28	Mild mosaic, mild yellow patches	8/20	8/20	40
	A + B + β	25	Mild mosaic, mild yellow patches	12/20	12/20	60
	A	-	-	0/20	0/20	0
	Mock inoculated control	-	-	0/20	0/20	0
Bitter gourd (cv. Co1)	A + B	25	Mild mosaic, mild leaf mottling	6/20	6/20	30
	A + B + β	22	Mild leaf curling, stunting	17/20	17/20	85
	A	-	-	0/20	0/20	0
	Mock inoculated control	-	-	0/20	0/20	0
Muskmelon (cv. Dulce F1)	A + B	25	Mosaic, vein banding	10/20	8/20	50
	A + B + β	23	Mosaic, vein banding, Downward leaf curling, stunting	15/20	15/20	75
	A	-	-	0/20	0/20	0
	Mock inoculated control	-	-	0/20	0/20	0
Cucumber (cv. Nazia F1)	A + B	25	Vein banding, yellowing	12/20	12/20	60
	A + B + β	23	Vein banding, yellowing	13/20	13/20	65
	A	-	-	0/20	0/20	0
	Mock inoculated control	-	-	0/20	0/20	0
Watermelon (cv. F1 555)	A + B	21	Mosaic, leaf mottling, yellowing	12/20	12/20	60
	A + B + β	20	Mosaic, leaf mottling, yellowing, stunting	17/20	17/20	85
	A	-	-	0/20	0/20	0
	Mock inoculated control	-	-	0/20	0/20	0
Bottle gourd (cv. Co1)	A + B	28	Mild mosaic	2/20	2/20	10
	A + B + β	24	Mosaic, yellow patches	9/20	9/20	45
	A	-	-	0/20	0/20	0
	Mock inoculated control	-	-	0/2	0/20	0

### 3.5. Viral DNA accumulation in the agro-inoculated cucurbits

**Figure 5:**
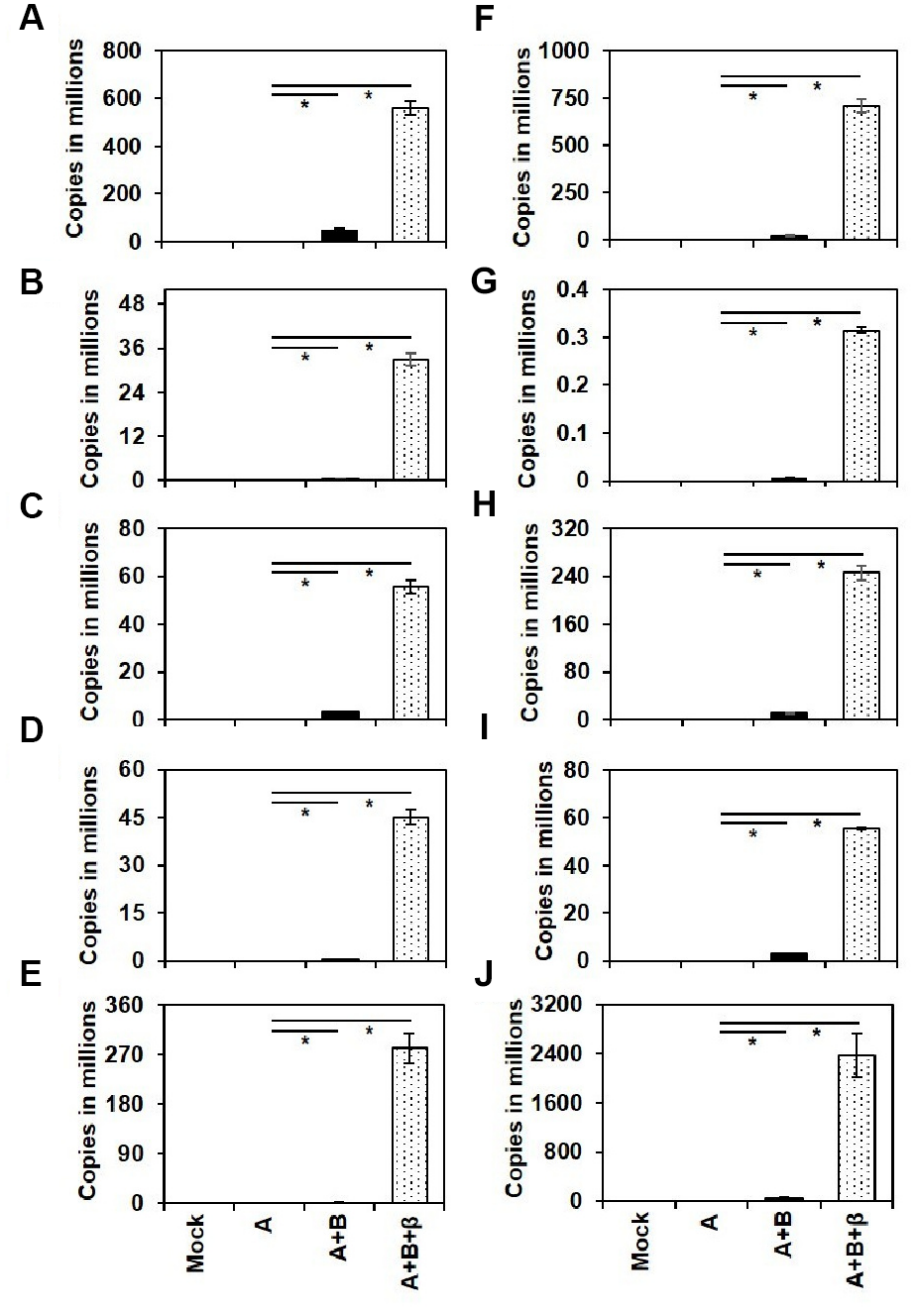
Viral DNA accumulation in the systemic leaves of the agro-inoculated cucurbits at 28 dpi. The cucurbit plants used are **(A)** ash gourd, **(B)** bottle gourd, **(C)** bitter gourd, **(D)** cucumber, **(E)** muskmelon, **(F)** pumpkin, **(G)** ridge gourd, **(H)** snake gourd, **(I)** squash and **(J)** watermelon. Each bar represents the mean of three individual replicates ± standard error and asterisks indicate statistical significance (*p* < 0.001) according to ANOVA *F*-test.

The presence of ToLCNDV genomic components and ToLCBB in the agro-inoculated plants were checked by conventional PCR using degenerate primers (Table 3). Further, qPCR using virus genome specific primers was employed to quantify the accumulation of ToLCNDV and ToLCBB in the systemic leaves of agro-inoculated plants. No viral DNA was detected in the mock or DNA-A alone inoculated plants whereas virus titer was detected in the co-inoculated plants at 28 dpi. A significant increase in the accumulation of DNA-A was observed in the co-inoculated plants of DNA-A + DNA-B and DNA-A + DNA-B + ToLCBB (Figure 5). Among these two treatments, the higher accumulation was observed in DNA-A + DNA-B + ToLCBB inoculated plants over DNA-A + DNA-B inoculated plants. Similar results were obtained in all inoculated cucurbits irrespective of the plant species tested.

## 4. Discussion

Among plant-infecting viruses, members of geminiviruses have emerged as a serious threat to cucurbit production globally. Initially, ToLCNDV was infecting tomato crops in India (Nagendran et al., 2019; Chakraborty and Kumar, 2021), but in recent decades it has widely expanded its host range to various vegetable crops such as chili (Kumar et al., 2015), okra (Venkataravanappa et al., 2012), potato (Jeevalatha et al., 2018), eggplant (Pratap et al., 2011), and several cucurbitaceous crops (Ito et al., 2008; Tiwari et al., 2010; Yamamoto et al., 2021; Kumari et al., 2022; Krishnan et al., 2023). Previously, the association of ToLCNDV with yellow stunt disease of ash gourd was reported from Northern India (Roy et al., 2013). Among various begomovirus species infecting cucurbits (Ito et al., 2008; Tiwari et al., 2010; Nagendran et al., 2017a; Sangeetha et al., 2018; Yamamoto et al., 2021; Kumari et al., 2022; Krishnan et al., 2023), Squash leaf curl China virus (SLCCNV) was reported with leaf curl and mosaic disease of ash gourd in India and Thailand, respectively (Sawangjit, 2009; Riyaz et al., 2013). Similarly, Samretwanich et al. (2000) have reported on the association of ToLCNDV with leaf yellowing disease of ash gourd in Thailand. In the present study, the association of ToLCNDV with this disease was characterized in ash gourd. The Koch’s postulates for this disease complex on various cucurbitaceous crops were satisfied. Furthermore, the influence of cognate DNA-B and non-cognate betasatellite on the disease development were also demonstrated.

The bipartite begomovirus (SLCCNV and ToLCNDV) complexes are associated with ash gourd diseases in India and Thailand (Samretwanich et al., 2000; Riyaz et al., 2013; Roy et al., 2013). Sequence analysis of the cloned genomes suggested the association of ToLCNDV with the mosaic disease of ash gourd in the Udumalpet region, India. Furthermore, several studies have documented the frequent association of ToLCNDV with mosaic disease of various cucurbits (excluding ash gourd) such as *Coccinia grandis, Cucumis melo, Cucurbita pepo, Luffa cylindrica, Momordica charantia, Sechuimedule* and *Trichosanthes cucumerina*, worldwide (Kumari et al., 2022). However, our study is the first report on the association of a begomovirus (ToLCNDV) with the mosaic disease of ash gourd. The cucurbit growing period likely coincides with the major tomato cultivation season, thus the potential spread of ToLCNDV from tomato to ash gourd cannot be ignored. This necessitates investigating the distribution of ToLCNDV across major ash gourd growing regions in the country. Collectively, the available evidence of such host range expansion by ToLCNDV to diverse plant families makes it one of the most devastating plant pathogens across the continents.

Recombination is a common phenomenon that occurs within and between virus species, and it plays a crucial role in the emergence and evolution of begomoviruses (Prasanna and Rai, 2007; Duffy and Holmes, 2008; George et al., 2015; Kumar and Chakraborty, 2018). In the present study, intraspecies recombination events were detected in the intergenic (IR), AC1, and BC1 regions. This result corroborates previous findings on these regions being recombination hotspots (Lefeuvre et al., 2007; Prasanna and Rai, 2007; George et al., 2015; Kumar et al., 2015, 2017). Moreover, the recombination event encompassing IR might have facilitated this ToLCNDV isolate in expanding its host range to ash gourd.

Koch’s postulates were confirmed for this disease complex on various cucurbitaceous crops including ash gourd. The DNA-A alone inoculated cucurbits were symptomless and no DNA-A was detected in the systemic leaves, whereas in the presence of cognate DNA-B component, mild symptoms were observed. This result highlights that this ToLCNDV isolate is a true bipartite begomovirus which requires DNA-B for its systemic movement in the tested cucurbitaceous crops. Our infectivity data is in agreement with previous findings of the indispensable nature of the DNA-B component in the pathogenicity of ToLCNDV (Padidam et al., 1995; Ranjan et al., 2014; Sangeeta Kumar et al., 2023). However, the cucurbits co-inoculated with DNA-A, DNA-B (cognate) and betasatellite (non-cognate) have developed severe symptoms with reduced latent period and multi-fold increased accumulation of DNA-A component (Figure 5; Table 3). This finding is consistent with the available literature, which demonstrated that the presence of betasatellite has resulted in enhanced symptom severity of ToLCNDV in tomato (Sivalingam and Varma, 2012; Jyothsna et al., 2013; Sangeeta Kumar et al., 2023). This data underscores the necessity of investigating the presence of betasatellite(s) with such disease complexes on cucurbitaceous crops. As this virus complex causes disease in various cucurbits, it can be utilized in identifying virus resistance genotypes among these cucurbitaceous crops.

## 5. Conclusion

The present study reports on the association of a bipartite begomovirus ToLCNDV with mosaic disease of ash gourd in India. The cloned genomic components were recombinant in nature. Furthermore, these cloned virus components cause disease in several cucurbitaceous crops including ash gourd, thereby fulfilling Koch’s postulates. Inoculation of DNA-A alone did not cause disease on cucurbits; however, the presence of either cognate DNA-B or non-cognate betasatellite resulted in enhanced symptom severity and helped begomovirus accumulation.

## Data availability statement

The datasets presented in this study can be found in online repositories. The names of the repository/repositories and accession number(s) can be found in the article/Supplementary material.

## Author contributions

SV: Formal analysis, Methodology, Writing – original draft. PR: Methodology, Supervision, Writing – review & editing. KN: Methodology, Writing – review & editing, Formal analysis, Validation. NS: Supervision, Validation, Writing – review & editing. RK: Data curation, Formal analysis, Validation, Writing – review & editing. RS: Supervision, Validation, Writing – review & editing. TB: Supervision, Writing – review & editing. GK: Conceptualization, Data curation, Formal analysis, Methodology, Resources, Validation, Writing – review & editing.
