# Supplementary material for: A distinct strain of tomato leaf curl New Delhi virus that causes mosaic disease in ash gourd and other cucurbitaceous crops
Source: Front Microbiol. 2023 Oct 26;14:1268333. doi: 10.3389/fmicb.2023.1268333 (PMC10641021; doi:10.3389/fmicb.2023.1268333)
Supplement: Supplementary file 1 [file Data_Sheet_1.docx]

**Supplementary Table 1. Sequence identity matrix based on DNA A complete genome and its ORFs of ToLCNDV ash gourd isolate with other ToLCNDV isolates reported previously**

| **Virus isolate** | **DNA A UDU1 (MZ073374)** | | | | | | | |
| --- | --- | --- | --- | --- | --- | --- | --- | --- |
|  | Accession No | DNA A | AV1 | AV2 | AC1 | AC2 | AC3 | AC4 |
| ToLCNDV:4 | KF551592 | 81.4 | 85.7 | 82.0 | 77.9 | 84.7 | 84.9 | 84.1 |
| ToLCNDV:5 | EF450316 | 88.3 | 89.6 | 90.0 | 88.3 | 85.8 | 85.6 | 90.9 |
| ToLCNDV:sev | U150152 | 90.6 | 93.2 | 93.2 | 90.6 | 86.8 | 86.6 | 93.7 |
| Chilli:Pak | OM102558 | 90.4 | 92.8 | 92.9 | 90.5 | 86.8 | 86.3 | 92.0 |
| Pum:India | JN129254 | 90.5 | 92.8 | 92.3 | 91.0 | 86.8 | 86.3 | 93.7 |
| Melon: Spain | MH577751 | 91.8 | 94.5 | 96.4 | 90.8 | 92.1 | 88.8 | 93.7 |
| Sp.Gourd:Pak | AM292302 | 90.1 | 93.1 | 92.9 | 90.1 | 87.5 | 86.8 | 92.6 |
| Tom:Pak | AM947506 | 90.2 | 92.2 | 90.2 | 90.8 | 85.8 | 84.9 | 93.2 |
| Brinjal:India | HQ264185 | 90.7 | 92.4 | 93.2 | 92.4 | 84.7 | 84.6 | 96.1 |
| Okra:India | EF035482 | 91.2 | 92.7 | 92.6 | 92.5 | 85.8 | 85.8 | 95.0 |
| A.gourd:India | JN208136 | 90.3 | 93.1 | 92.3 | 90.9 | 86.1 | 85.1 | 92.6 |
| B.G:Thailand | AB368447 | 89.5 | 92.2 | 93.5 | 89.4 | 88.6 | 87.1 | 94.9 |
| Tom:Bang | KM383744 | 89.9 | 92.8 | 93.2 | 89.9 | 88.2 | 86.6 | 94.3 |
| Cu:Indonesia | AB613825 | 88.6 | 92.0 | 92.0 | 87.0 | 88.6 | 86.6 | 92.3 |
| B.gourd:India | MW620975 | 83.8 | 92.6 | 95.3 | 77.3 | 83.6 | 83.6 | 67.7 |
| Cu:India | KC545812 | 90.5 | 92.9 | 93.5 | 91.4 | 85.4 | 85.1 | 95.4 |
| Sp.Gourd:India | MH475911 | 90.5 | 92.9 | 93.5 | 91.4 | 86.1 | 85.4 | 94.9 |
| Potato:India | AY286316 | 89.9 | 92.0 | 91.4 | 91.0 | 85.8 | 84.6 | 96.1 |
| Tom:India | AY428769 | 90.1 | 92.7 | 93.2 | **92.6** | 87.5 | 88.5 | 94.3 |
| Chilli:India | EU309045 | 90.9 | 92.6 | 92.9 | 92.0 | 86.8 | 85.8 | 94.9 |
| Bo.gourd:India | MZ073372 | 91.1 | 92.9 | 93.2 | 92.1 | 86.5 | 85.4 | **96.6** |
| R.Gourd:India | KT426905 | **92.4** | **96.7** | **97.3** | 91.3 | **92.5** | **89.0** | 93.7 |
| Chrysan:India | MG758145 | 92.0 | 96.3 | 95.8 | 91.3 | 92.5 | 88.0 | 93.7 |
| ToLCNDV:2 | JQ897969 | 72.0 | 77.5 | 72.8 | 72.7 | 73.4 | 68.7 | 80.8 |
| Squash: Tunisia | MF967021 | 91.7 | 94.4 | 96.4 | 91.0 | 91.8 | 88.5 | 83.7 |
| Zucchini: Spain | MK279352 | 91.7 | 94.5 | 96.5 | 90.9 | 92.1 | 88.8 | 83.7 |

**Supplementary Table 2. Sequence identity matrix based on DNA B completegenome and its ORFs of ToLCNDV ash gourd isolate with other ToLCNDV isolates reported previously**

| **DNA-B UDU1 (MZ073373)** | | | | |
| --- | --- | --- | --- | --- |
| Virus isolate | Accession No | DNA-B | BV1 | BC1 |
| Pum:India | AM286435 | 81.3 | 85.6 | 88.8 |
| Cu:Thailand | AB330080 | 78.8 | 83.6 | 87.2 |
| Jes:Svr:BG | AJ875158 | 78.6 | 83.0 | 86.5 |
| B.G:India | MW620976 | 80.6 | 85.7 | 86.0 |
| NDV:India | AY438563 | 79.7 | 83.1 | 86.0 |
| C.roseus:Pak | MT085664 | 78.5 | 81.1 | 86.5 |
| Chilli:Pak | DQ116882 | 81.3 | 86.6 | 87.4 |
| Potato:India | EF043233 | 80.4 | 85.3 | 88.1 |
| Potato:India | KC874497 | 81.0 | 85.7 | 88.4 |
| Tom:Pak | AY150304 | 80.5 | 85.1 | 88.2 |
| B.Gourd:India | DQ020490 | 81.2 | 84.8 | 88.7 |
| BYVMV:Ind | HQ586007 | 81.6 | 86.3 | 88.4 |
| w.melon_Ind | MK087117 | 78.3 | 86.4 | 87.4 |
| Tom:India | HQ141674 | 80.8 | 85.6 | 88.5 |
| Cu:India | KC545813 | 82.2 | 86.7 | 88.9 |
| NDV:India | DQ169057 | 82.3 | 86.9 | 89.2 |
| Sp.G:India | AY939924 | 81.9 | 86.7 | 89.2 |
| RG:India | HM989846 | 82.2 | 86.9 | 89.2 |
| Zuc:Spain | MH577612 | **96.1** | 97.5 | 98.3 |
| Tom:spain | KM977734 | 96.0 | **97.6** | **98.5** |
| B.G:Erd:Ind | MW620978 | 80.5 | 85.7 | 86.0 |
| TLCPV:Ind | AM992534 | 71.3 | 74.1 | 82.3 |
| TLCPV:Iran | FJ660443 | 70.9 | 73.5 | 81.8 |
