# Supplementary figures and images for: A distinct strain of tomato leaf curl New Delhi virus that causes mosaic disease in ash gourd and other cucurbitaceous crops
Source: Front Microbiol. 2023 Oct 26;14:1268333. doi: 10.3389/fmicb.2023.1268333 (PMC10641021; doi:10.3389/fmicb.2023.1268333)

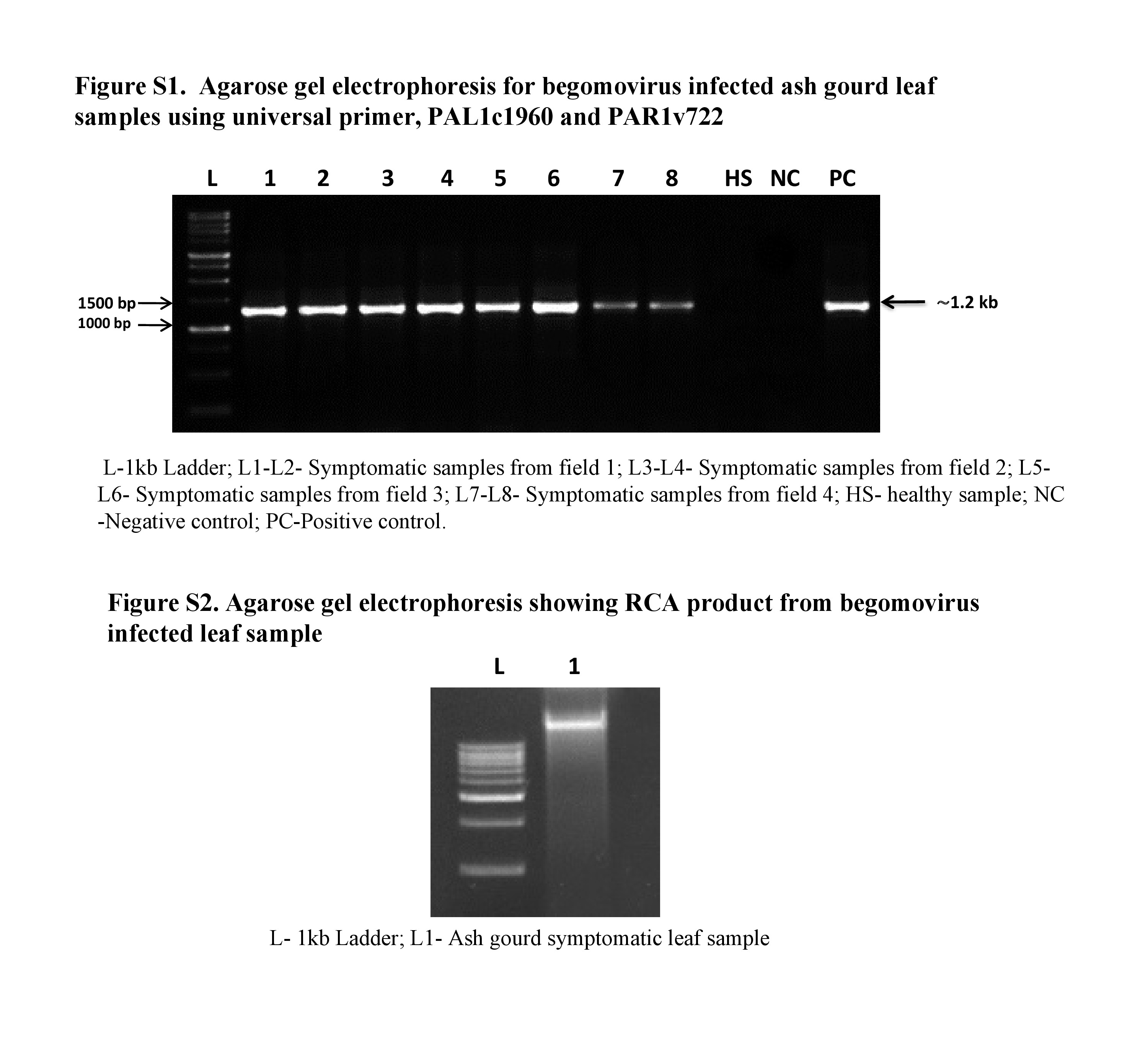

Supplement: Supplementary file 2 [file Image_1.JPEG]

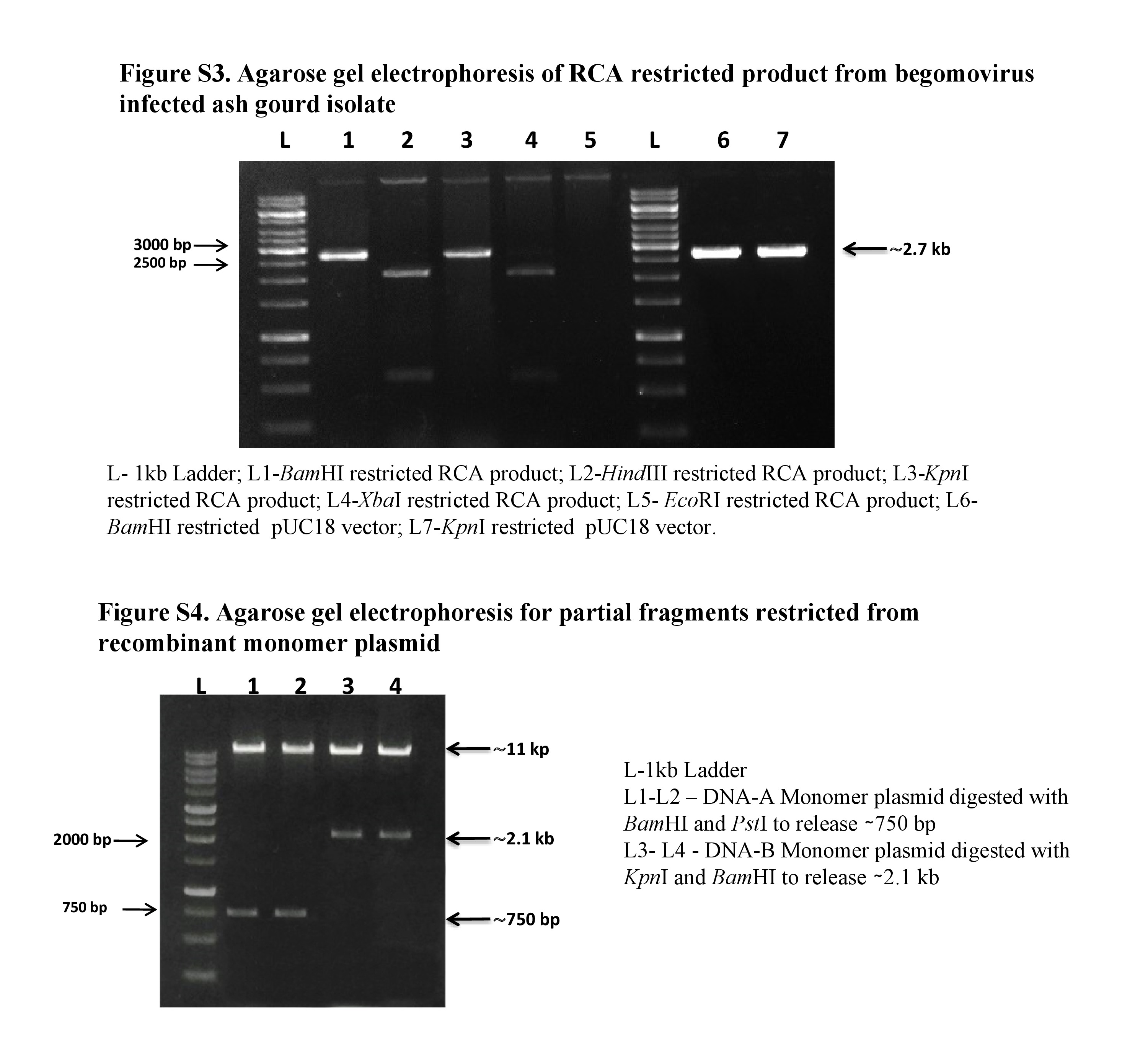

Supplement: Supplementary file 3 [file Image_2.JPEG]

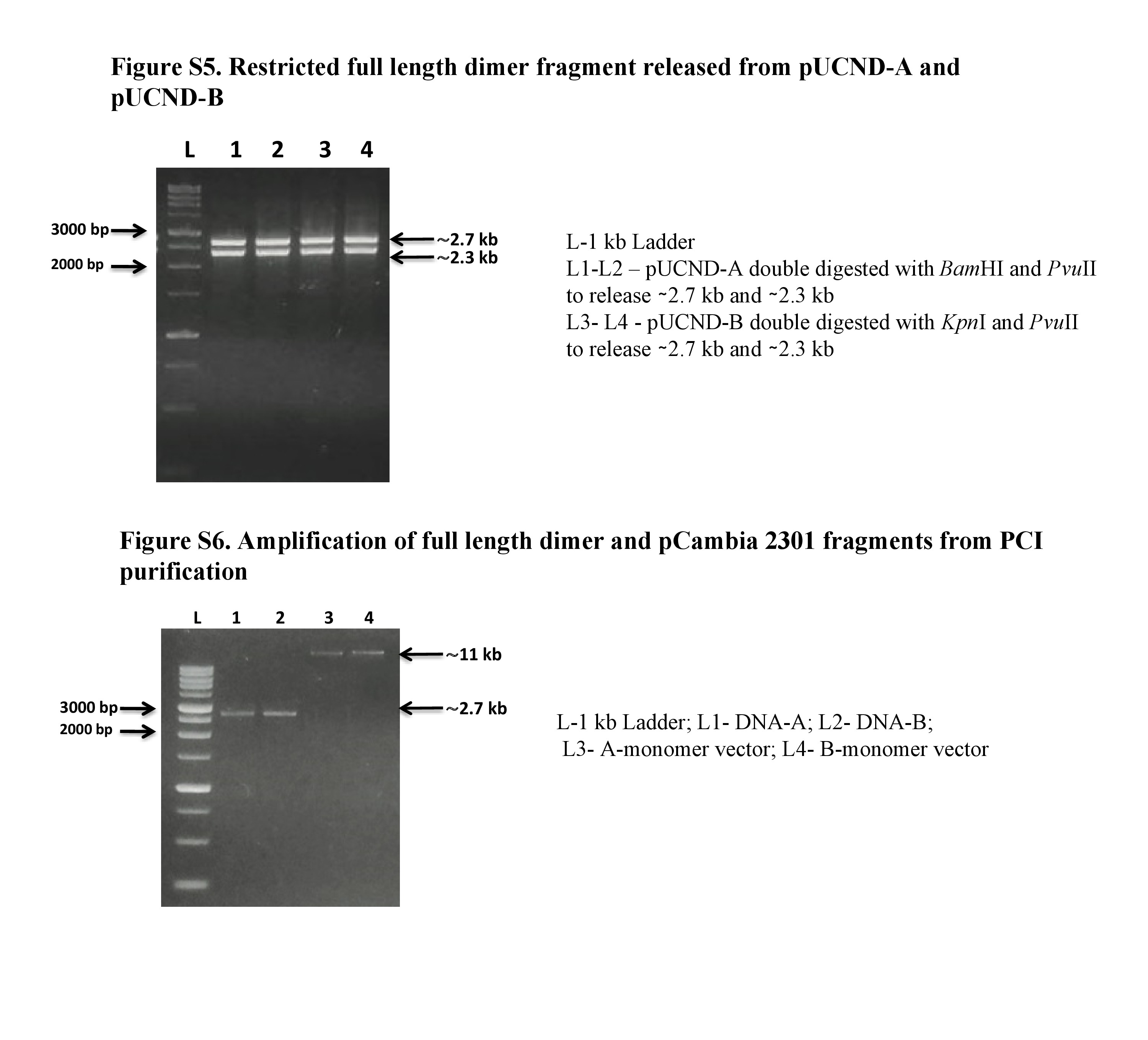

Supplement: Supplementary file 4 [file Image_3.JPEG]

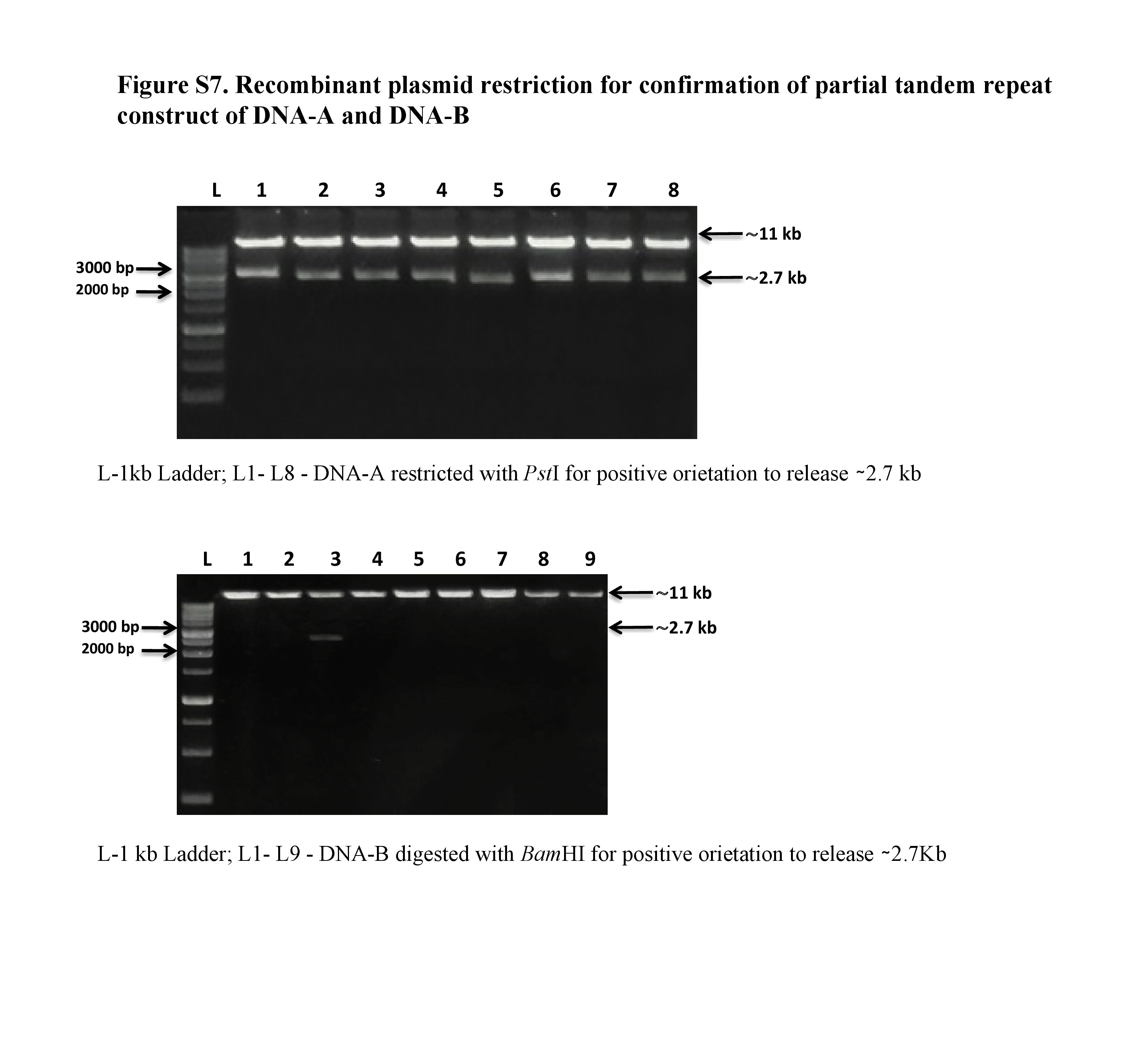

Supplement: Supplementary file 5 [file Image_4.JPEG]

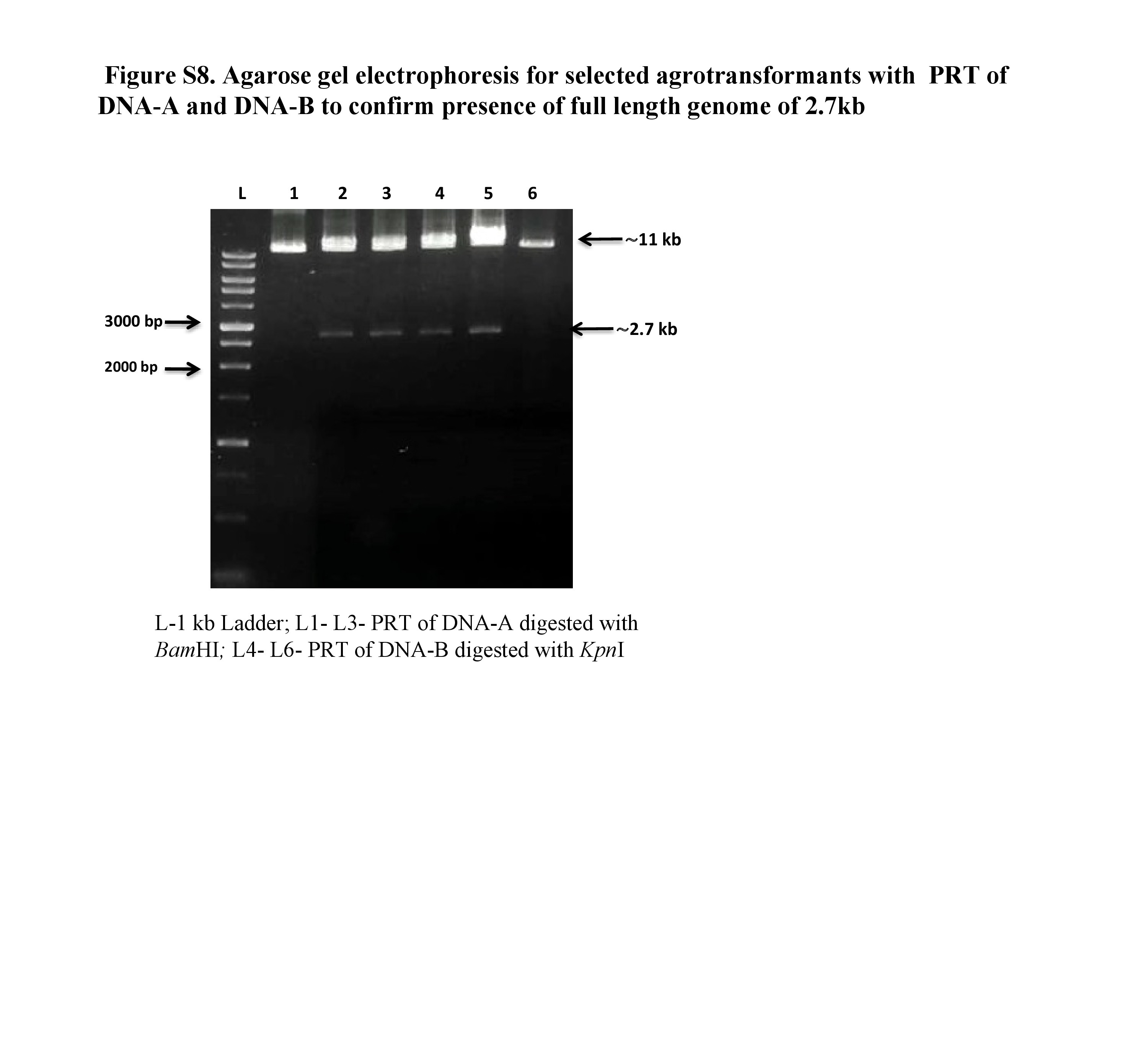

Supplement: Supplementary file 6 [file Image_5.JPEG]

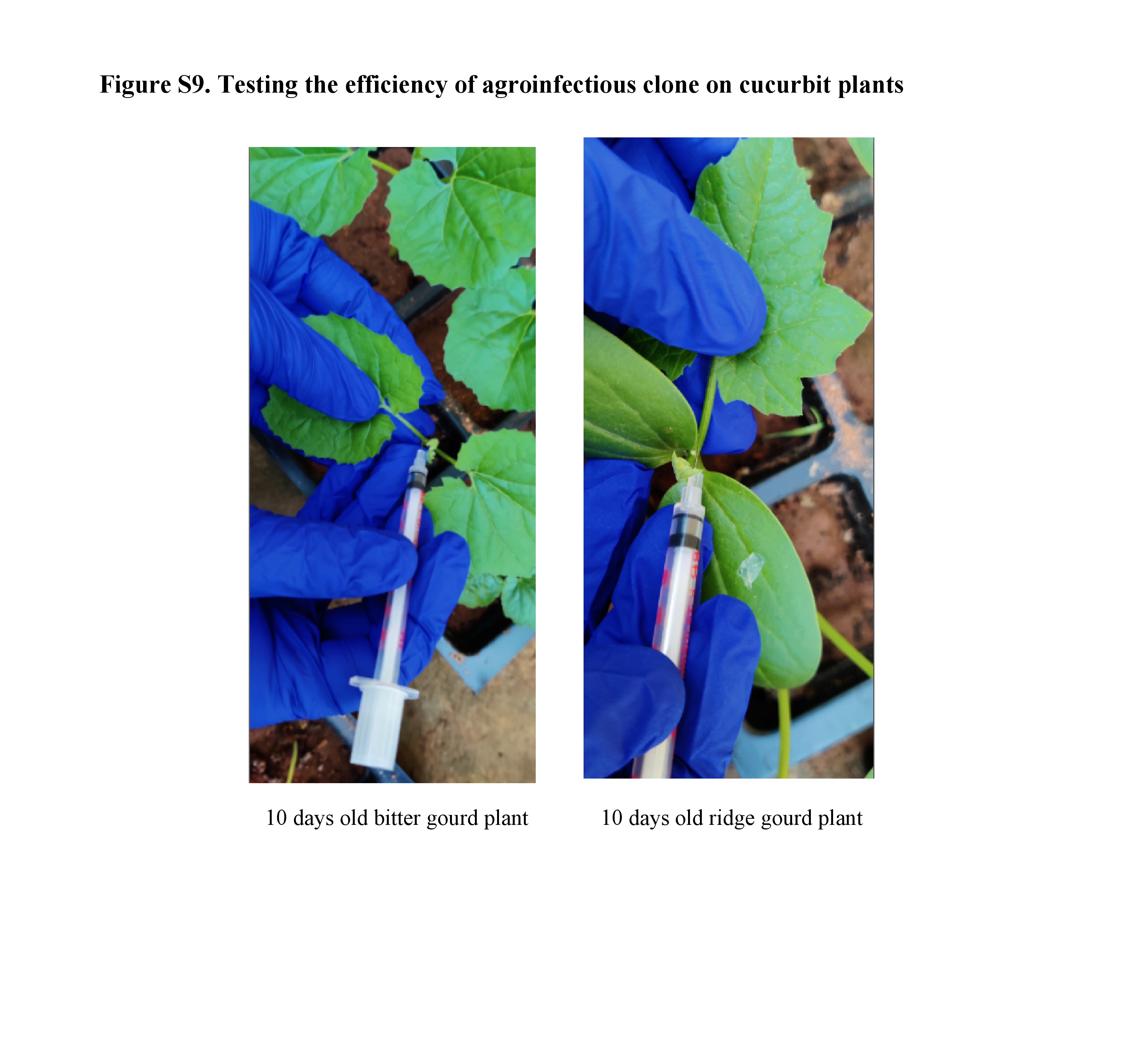

Supplement: Supplementary file 7 [file Image_6.JPEG]

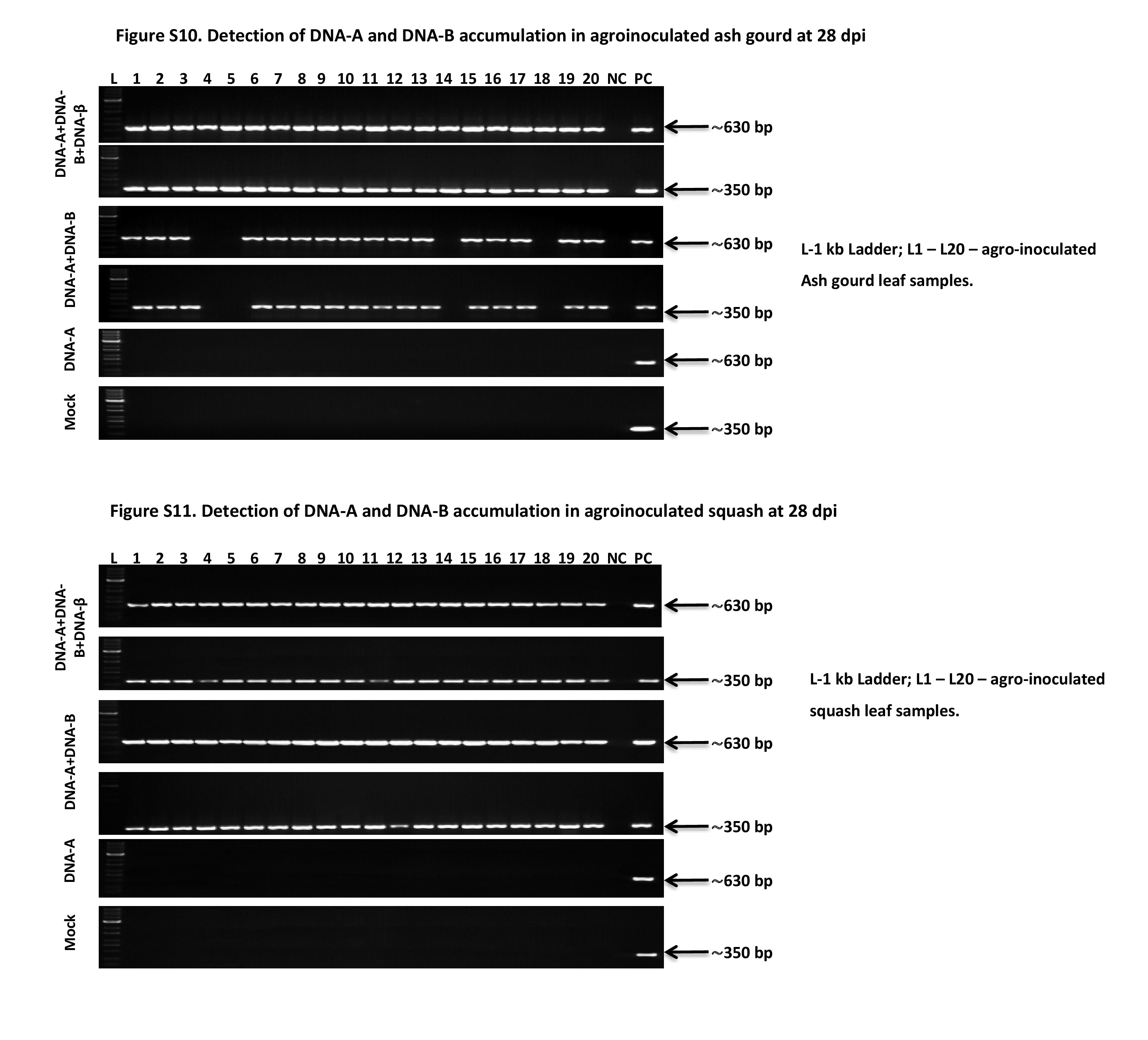

Supplement: Supplementary file 8 [file Image_7.JPEG]

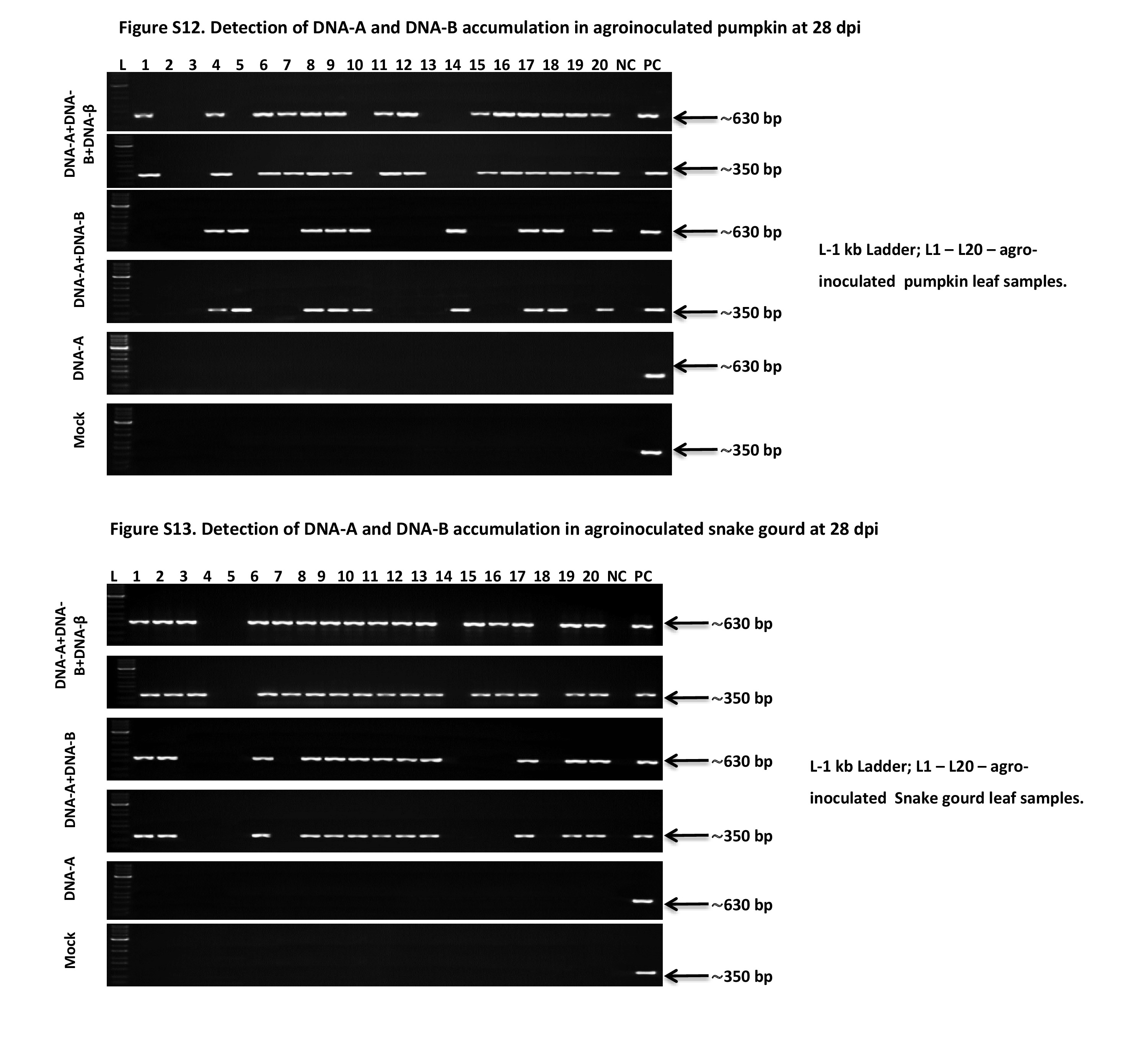

Supplement: Supplementary file 9 [file Image_8.JPEG]

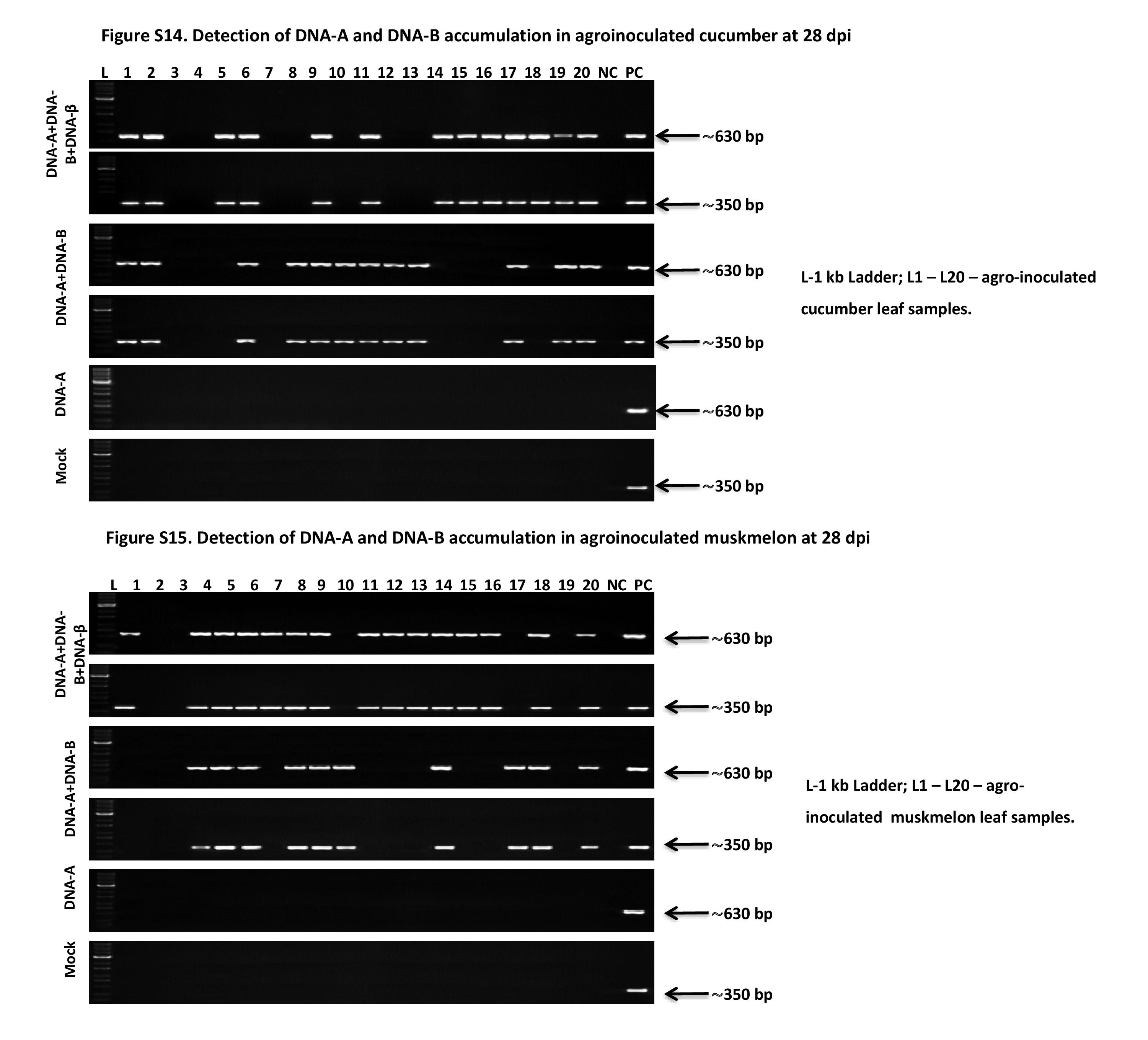

Supplement: Supplementary file 10 [file Image_9.JPEG]

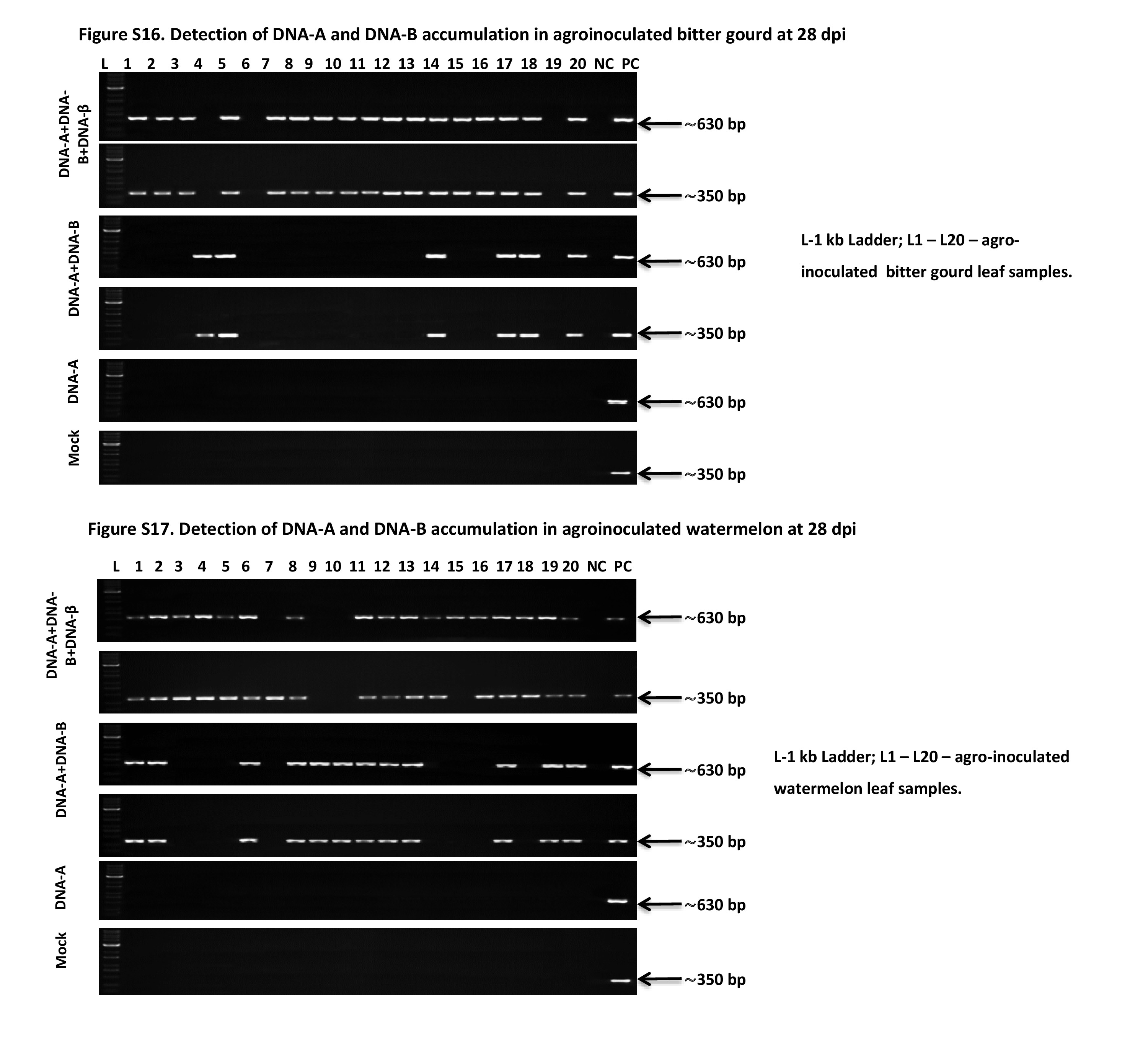

Supplement: Supplementary file 11 [file Image_10.JPEG]

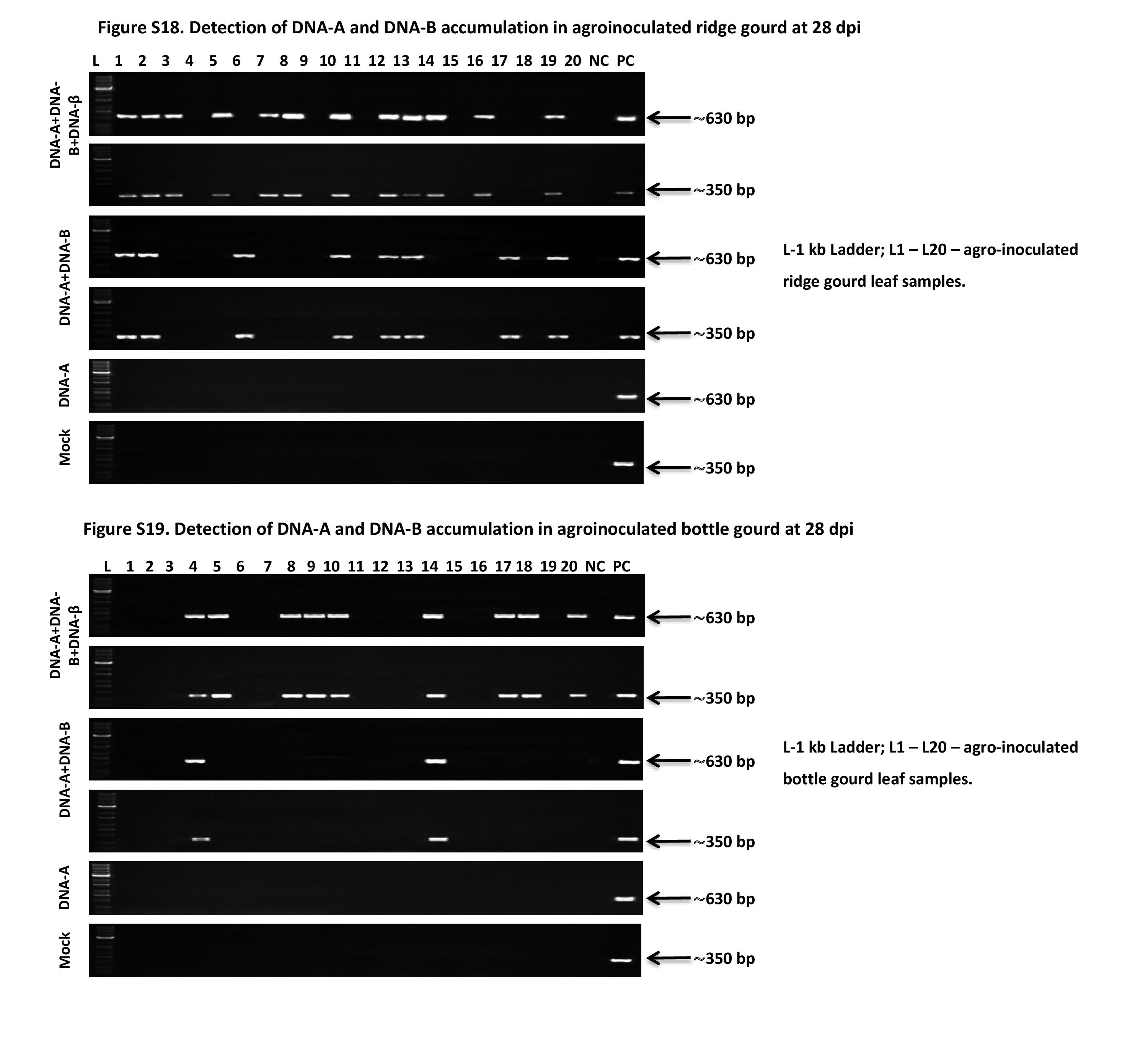

Supplement: Supplementary file 12 [file Image_11.JPEG]
